# Perfusion imaging of neuroblastoma and nephroblastoma in a paediatric population using pseudo-continuous arterial spin-labelling magnetic resonance imaging

**DOI:** 10.1007/s10334-021-00943-y

**Published:** 2021-08-03

**Authors:** Anita Adriaantje Harteveld, Annemieke Simone Littooij, Max Maria van Noesel, Marijn van Stralen, Clemens Bos

**Affiliations:** 1 Department of Radiology, University Medical Centre Utrecht, Utrecht University, P.O. box 85500, 3508 GA Utrecht, The Netherlands; 2 Department of Radiology and Nuclear Medicine, Erasmus MC University Medical Centre Rotterdam, Rotterdam, The Netherlands; 3 Princess Máxima Centre for Paediatric Oncology, Utrecht, The Netherlands

**Keywords:** Arterial spin labelling (ASL), Magnetic resonance imaging, Perfusion, Paediatrics, Neoplasms

## Abstract

**Objectives:**

To examine the feasibility of performing ASL-MRI in paediatric patients with solid abdominal tumours.

**Methods:**

Multi-delay ASL data sets were acquired in ten paediatric patients diagnosed with either a neuroblastoma (*n* = 4) or nephroblastoma (*n* = 6) during a diagnostic MRI examination at a single visit (*n* = 4 at initial staging, *n* = 2 neuroblastoma and *n* = 2 nephroblastoma patients; *n* = 6 during follow-up, *n* = 2 neuroblastoma and *n* = 4 nephroblastoma patients). Visual evaluation and region-of-interest (ROI) analyses were performed on the processed perfusion-weighted images to assess ASL perfusion signal dynamics in the whole tumour, contralateral kidney, and tumour sub-regions with/without contrast enhancement.

**Results:**

The majority of the included abdominal tumours presented with relatively low perfusion-weighted signal (PWS), especially compared with the highly perfused kidneys. Within the tumours, regions with high PWS were observed which, at short PLD, are possibly related to labelled blood inside vessels and at long PLD, reflect labelled blood accumulating inside tumour tissue over time. Conversely, comparison of ASL perfusion-weighted image findings with T_1_w enhancement after contrast administration showed that regions lacking contrast enhancement also were void of PWS.

**Discussion:**

This pilot study demonstrates the feasibility of utilizing ASL-MRI in paediatric patients with solid abdominal tumours and provides a basis for further research on non-invasive perfusion measurements in this study population.

## Introduction

In paediatric oncology, imaging plays a defining role in the initial diagnosis, staging, early post-treatment response assessment and surveillance. Magnetic resonance imaging (MRI) is currently the preferred modality for clinical assessment of tumours located in the abdomen [1]. Clinicians rely mainly on anatomical imaging techniques for staging, risk stratification and assessing treatment response [2]. The state-of-the-art method to evaluate response to treatment in solid tumours is by measuring the tumour size following the RECIST (Response Evaluation Criteria in Solid Tumours) guidelines [3–5]. In addition to anatomic assessment, functional measures such as perfusion and diffusion may provide complementary information for the clinical assessment of tumour diagnosis, staging, and treatment response [4, 6–9].

The characterisation of tumour hemodynamics in children is still limited. One explanation is that childhood cancers are very heterogeneous with often relatively small patient numbers, limiting standardisation for imaging modality choice and subsequent technical optimisation [1].

Arterial spin-labelling (ASL) MRI may be a valuable method in a paediatric population for non-invasive measurement of tissue perfusion without the need for contrast administration. This method utilises magnetically labelled water-protons in blood as an endogenous tracer. This allows for repeated measurements within the same scan session or frequent follow-up MRI examinations. Thus far, in paediatric patients ASL-MRI has been performed in a few studies for perfusion imaging of brain tumours [10–14]. These studies showed that ASL perfusion signal is correlated with tumour histopathological vascular density and can be used to distinguish high- and low-grade tumours. In addition, ASL renders characteristic perfusion patterns for diverse pathologic types of brain tumours in children. These findings suggest an important value of ASL-MRI in the characterisation of hemodynamics in brain tumours. This has not yet been studied in paediatric abdominal tumours.

Therefore, the purpose of this study was to examine the feasibility of performing ASL-MRI in paediatric patients with tumours located outside the brain by evaluating the presentation of ASL perfusion signal dynamics of common abdominal tumours.

## Materials and methods

### Study population

This is a report on retrospective, cross-sectional data obtained in patients who received diagnostic MR imaging of the abdomen related to an abdominal solid tumour. An ASL scan had been added to the clinical scan protocol in consultation with the pediatric radiologist for the assessment of tumour perfusion. Subjects did not have to follow specific actions or rules for conducting this study, since the patients were scheduled for the MRI examination as part of the clinical diagnostic procedures. A letter waiving ethical approval was obtained from the IRB of our institution. The image data were fully anonymised before processing and analysis.

### MR Imaging

All patients were scanned on a 1.5 T MR system (Ingenia, Philips, Best, the Netherlands; software release 5.4) equipped with a body coil for transmission and a 28-element phased array coil for reception.

#### ASL scan

The ASL scan was acquired using pseudo-continuous ASL (pCASL) labelling [15], as implemented by the vendor and clinically available as a standard sequence, with three different post-labelling delays (PLD; 0.5, 1.0, and 1.5 s) and a label duration of 1.5 s [16]. An auxiliary sequence was acquired to estimate equilibrium magnetisation (M_0_). Each PLD was obtained in a separate acquisition that consisted of 10 label-control pairs. The M_0_ scan, essentially the pCASL scan without labelling, was acquired four times and averaged after motion correction. All scans were performed with the same 2D multi-slice image readout using single-shot gradient-echo EPI. The labelling slab and image readout were planned exactly the same for each ASL acquisition. The applied scan parameters are provided in Table 1. Total acquisition time of the ASL and M_0_ scans was 4min54s.

**Table 1:** Scan parameters

Parameters	ASL	M_0_^a^
TR/TE [ms]	4000/21	6000/21
EPI-factor	55	
Flip angle [deg]	90	
SENSE	1.5 (RL direction)	
FOV [mm^2^]	244 × 244	
Acquired voxel size [mm^2^]	3 × 3	
Slice thickness [mm]	6	
Slice gap [mm]	1	
No. of slices	9	
Phase encoding direction	RL	
Fold-over suppression	Saturation slabs^b^	
Fat-suppression	SPIR	
Slice orientation	Coronal	
Slice scan order	AP	
No. of repetitions	10^c^	4
Labelling duration [s]	1.5	N/A
Delay time [s]	0.5, 1.0, 1.5	N/A
Total acquisition time [min:s]	01:28 (per delay time)	00:30

*AP* anterior–posterior, *ASL* arterial spin labelling, *EPI* echo planar imaging, *FH* feet–head, *FOV* field-of-view, *SENSE* sensitivity encoding, *SPIR* spectral presaturation with inversion recovery, *TE* echo time, *TR* repetition time

^a^Only parameter settings that were different from the ASL scan

^b^Spatial saturation slabs superior and inferior to the image volume to suppress undesired signal aliasing

^c^Label-control pairs per delay time

The ASL and M_0_ scans were performed at the end of the MRI examination, before the injection of contrast agent. The localiser scan and subsequently acquired clinical scans were used to enable correct planning of the ASL and M_0_ scans. Slices were planned in coronal orientation to minimise through-plane motion due to breathing, including as much tumour tissue as possible and (part of) the contralateral kidney within the stack thickness (62 mm). The contralateral kidney was used as a reference organ for comparison with the ASL perfusion signal measured in the tumour. Normally, the kidneys are well perfused, and therefore, measured ASL perfusion signal in this organ could be used as an indication of successful labelling within a subject. The labelling slab of the ASL scan was placed perpendicular to the descending aorta and above the tumour (and kidneys) to prevent these tissues from sliding into the labelling slab during breathing. Care was taken that the labelling slab was as close as possible to the tumour and remained below the diaphragm (if possible) to minimise susceptibility artifacts near the lungs. B_0_ shimming was applied to the imaging stack and the labelling slab independently. Most patients were under anaesthesia during the entire MRI examination. Acquisitions were performed under non-invasive ventilation or free-breathing conditions in supine position.

#### Clinical scans

A standard clinical scan protocol was obtained in each subject, which included (among others) T_2_-weighted (T_2_w) and T_1_-weighted (T_1_w) scans. A 3D T_2_w TSE scan was acquired with TE 90 ms and isotropic spatial resolution 1.2 × 1.2 × 1.2 mm^3^. T_1_w gradient-echo imaging before and after contrast agent administration (CE-T_1_w) was performed either using the 3D THRIVE technique with fat suppression, TE 2.7 ms and acquired spatial resolution 1.07 × 1.07 × 3 mm^3^, or the 3D Vane XD technique with TE_1_/TE_2_ 1.9/4.0 ms (with Dixon-based water-fat separation) and acquired spatial resolution 1.5 × 1.5 × 3 mm^3^.

### Image processing

#### ASL scan

To compensate for misalignments caused by (respiratory) motion during image acquisition, image registration was performed using Elastix [17]. Since tissues and organs inside the abdominal region may move with respect to each other, each organ of interest (tumour, kidney) was registered separately. To this end, images were first cropped to the region of interest before image registration. The ASL data set was registered using a principal component-based groupwise method [18]. Multi-PLD raw ASL and M_0_ images were registered slicewise (total of 64 images per slice; 4 M_0_ repetitions, 10 label-control pairs per PLD). The groupwise registration method is robust against intensity changes between the images and does not require the choice of a reference image avoiding registration bias [18].

After image registration, label-control raw ASL images were pairwise subtracted. Next, outlier rejection was performed for each PLD by excluding subtraction images containing > 20% voxels with a value of more than ± 1.5 SD from the voxel wise mean value over all repetitions within the tumour or kidney region. The subtraction images remaining after outlier rejection were averaged per PLD to obtain averaged perfusion-weighted images ($\overline{{\Delta {\text{M}}}}$). The definition of the tumour and kidney masks that were used for the outlier rejection is described in more detail in the Image Analysis section.

#### Clinical scans

For assessment of contrast enhancement of the tumour, T_1_w subtraction images (T_1_w-subtracted) were used. To this end, first the CE-T_1_w scan was coregistered to the T_1_w scan using 3D rigid image registration in Elastix [17], and next both images were subtracted.

### Image analysis

Regions-of-interest (ROI) analyses were performed to quantitatively evaluate the measured ASL perfusion signal in different regions for each subject. ROIs were drawn manually around the tumour and contralateral kidney contours on the averaged M_0_ images (after image registration). The ROI of the contralateral kidney included the renal parenchyma. In addition, ROIs were drawn in enhancing and non-enhancing tumour subregions that were identified on T_1_w subtraction images. ROIs were drawn conservatively in the enhancing and non-enhancing tumour regions, i.e., well within the visible boundary of a non-enhancing or enhancing region, to take into account minor registration errors between the ASL perfusion and the CE-T_1_w data sets. The 3D anatomical T_2_w and T_1_w images were reformatted to the geometry of the M_0_ images to cross-check the tumour contours. When necessary, ROIs were manually adjusted with in-plane translations between anatomical and ASL scans. In case multiple tumours were present, the largest tumour was used for the ROI analysis. For each tissue type (whole-tumour, contralateral kidney, enhancing tumour, non-enhancing tumour) the same ROI was used within an individual patient. ROIs were drawn by a single observer (A.H) and were verified and corrected by a radiologist (A.L with > 10 years of experience with assessing paediatric oncology MR images).

ASL perfusion signal dynamics within the tumour and contralateral kidney were assessed by calculating the relative perfusion-weighted signal (PWS) for each delay time as $\overline{{\Delta {\text{M}}}} /{\text{M}}_{0} \times 100\%$. Mean PWS values were calculated over all voxels inside the ROIs for each subject.

Image processing and analysis were performed using custom scripts in MeVisLab (version 3.2; MeVis Medical Solutions AG, Bremen, Germany).

## Results

### Study population

ASL scans were obtained in 10 paediatric patients (mean age 4.3-year-old; range 1.7–8.3 years; 4 male) diagnosed with either a neuroblastoma (*n* = 4) or nephroblastoma (*n* = 6). In each patient, ASL imaging was performed at a single visit; *n* = 4 patients (*n* = 2 neuroblastoma, *n* = 2 nephroblastoma) at initial staging MRI examination pre-treatment, and *n* = 6 patients (*n* = 2 neuroblastoma, *n* = 4 nephroblastoma) at follow-up MRI-examination during treatment. Patient characteristics and primary tumour classification are presented in Table 2. Imaging, including ASL, was successfully performed in all subjects and used for further image processing and analysis.

**Table 2:** Baseline characteristics of the study population

Subject	Age (years)	Sex	Primary tumour type	Tumour location	Tumour stage [37]	Tumour size	Treatment pre-MRI
1	7.8	F	Neuroblastoma	Left adrenal gland with local and distant (supraclavicular) lymph node metastasis	INSS stage 4	Primary tumour: 3.8 × 2.3 × 3.1 cm; Lymph node metastases renal hilum: 4.0 × 2.5 × 6.3 cm	2 × N5/N6, 2 × N8, 2 × MIBG therapy, 2nd line chemotherapy:6 × Bevacizumab and Temozolomide
2	8.3	F	Nephroblastoma	Right kidney (upper pole and lower pole)	Upper pole: local stage I; Lower pole: local stage III (biopsy)	Upper pole: 1.8 × 1.7 × 1.2 cm; Lower pole: 3.0 × 3.9 × 2.5 cm	None. MRI performed at staging
3	4.9	M	Nephroblastoma	Bilateral (largest left kidney)	Left kidney: local stage III; Clinical stage V (bilateral disease with bone metastasis)	Left kidney: 10.0 × 7.4 × 7.6 cm	8 week pre-operative treatment 3 drugs: VCR/ACT/DOX
4	1.7	M	Neuroblastoma	Left adrenal gland with local and distant (supraclavicular) lymph node metastases	INSS stage 4	5.8 × 3.6 × 6.3 cm	3 × N5/N6
5	2.7	F	Nephroblastoma	Right kidney	Local stage II	7.4 × 6.1 × 6.0 cm	4 week chemo (VCR/ACT)
6	3.3	F	Nephroblastoma	Right kidney	Local stage I	7.3 × 6.9 × 6.4 cm	4 week chemo (VCR/ACT)
7	4.1	F	Neuroblastoma	Left adrenal gland with liver, bone marrow and lymph node metastases	INSS stage 4	10.9 × 8.8 × 8.5 cm	None. MRI performed at staging
8	3.8	M	Neuroblastoma	Right adrenal gland	INSS stage 3	12.7 × 10.8 × 11.2 cm	None. MRI performed at staging
9	2.2	F	Nephroblastoma	Right kidney	Local stage I	8.5 × 7.7 × 8.3 cm	4 week chemo (VCR/ACT)
10	4.1	M	Nephroblastoma	Right kidney	Local stage I	6.8 × 4.7 × 4.6 cm	None. MRI performed at staging

*ACT* actinomycin, *DOX* doxorubicin, *INSS* International Neuroblastoma Staging System, *MIBG* meta-iodobenzyl guanidine, *N5* cisplatin, etoposide and vindesine, *N6* vincristine, dacarbacine, ifosfamide and doxorubicin, *N8* topotecan, cyclophosphamide and etoposide, *VCR* vincristine

### Image processing and analysis

**Fig. 1:**
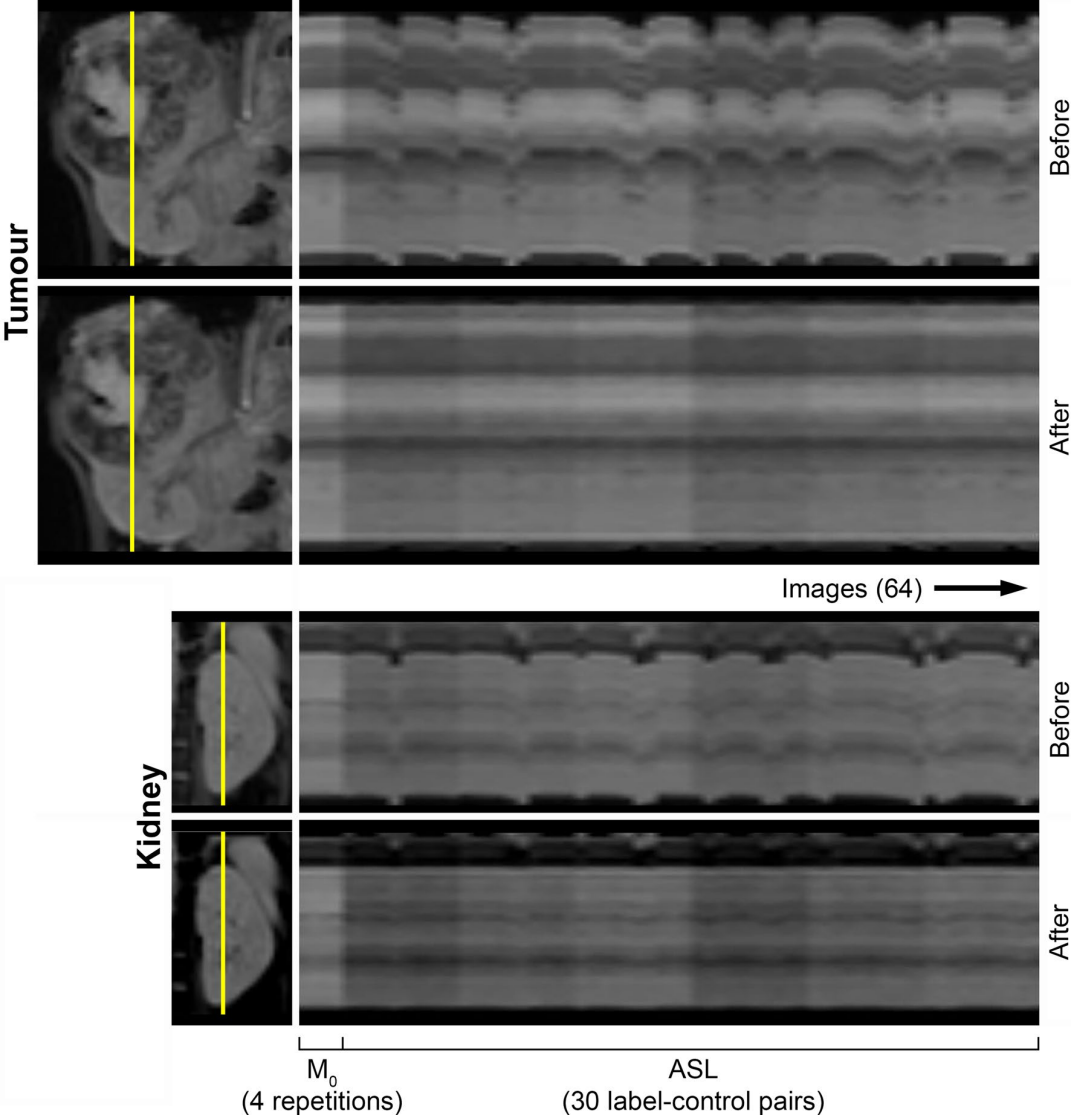
Example of motion correction results of images cropped around the tumour and contralateral left kidney (subject 8 in Table 2). Images visualise the same intersection (yellow line in the coronal image) over all M_0_ and raw ASL images. Improved alignment can be observed after image registration, for instance by looking at voxels representing the edges of the tumour/kidney

**Fig. 2:**
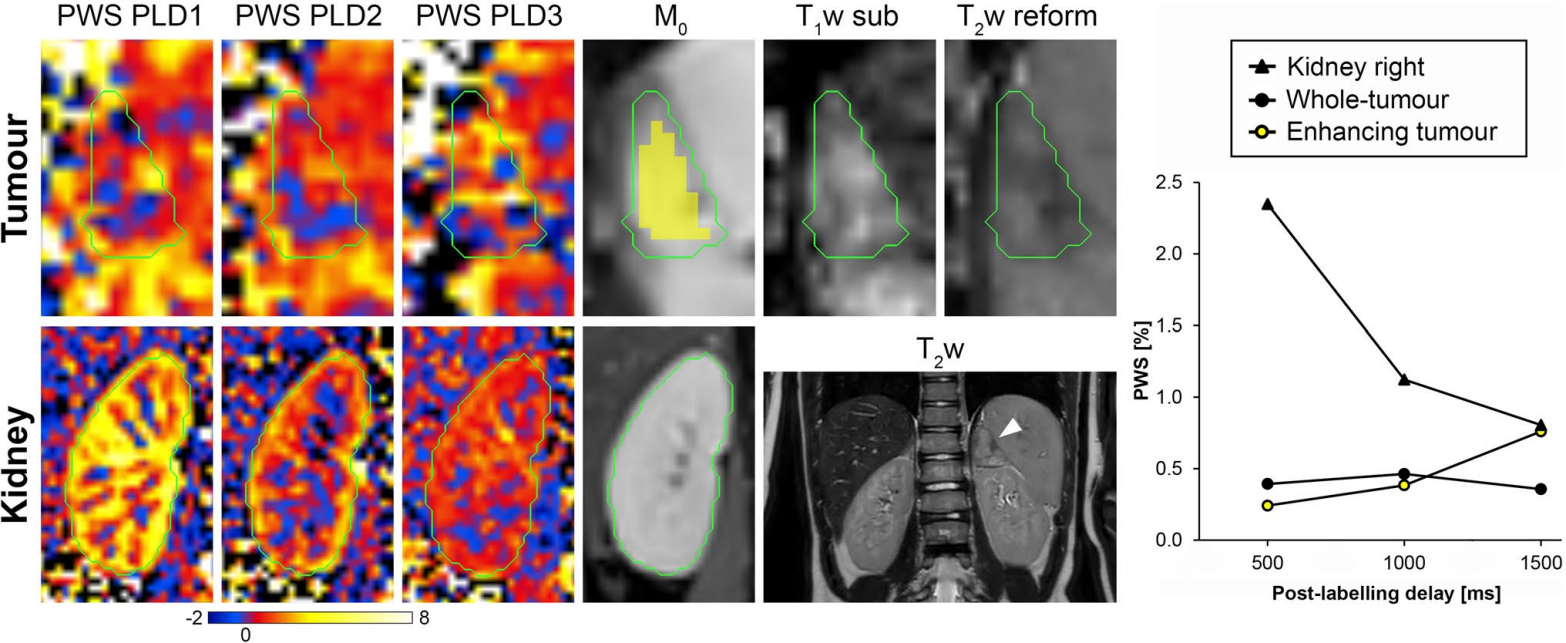
A 7-year-old female patient diagnosed with a neuroblastoma in the left adrenal gland (subject 1 in Table 2). Processed ASL and M_0_ images are shown cropped around the tumour (upper row) and right kidney (bottom row), together with the reformatted T_2_w and T_1_w-subtracted images that were used for analysis of the tumour. The anatomical location of the neuroblastoma can be appreciated on the coronal T_2_w images (arrow). The graph on the right shows the perfusion-weighted signal (PWS) as a function of post-labelling delay (PLD) resulting from the ROI analysis. Green contours: whole-tumour and kidney regions that were used for the ROI analysis; yellow ROI (overlayed on M_0_ image tumour): tumour region with contrast enhancement on the T_1_w-subtracted image. Colour scale bar indicates PWS (∆M/M_0_ × 100%). PLD1 = 0.5 s; PLD2 = 1.0 s; PLD3 = 1.5 s

**Fig. 3:**
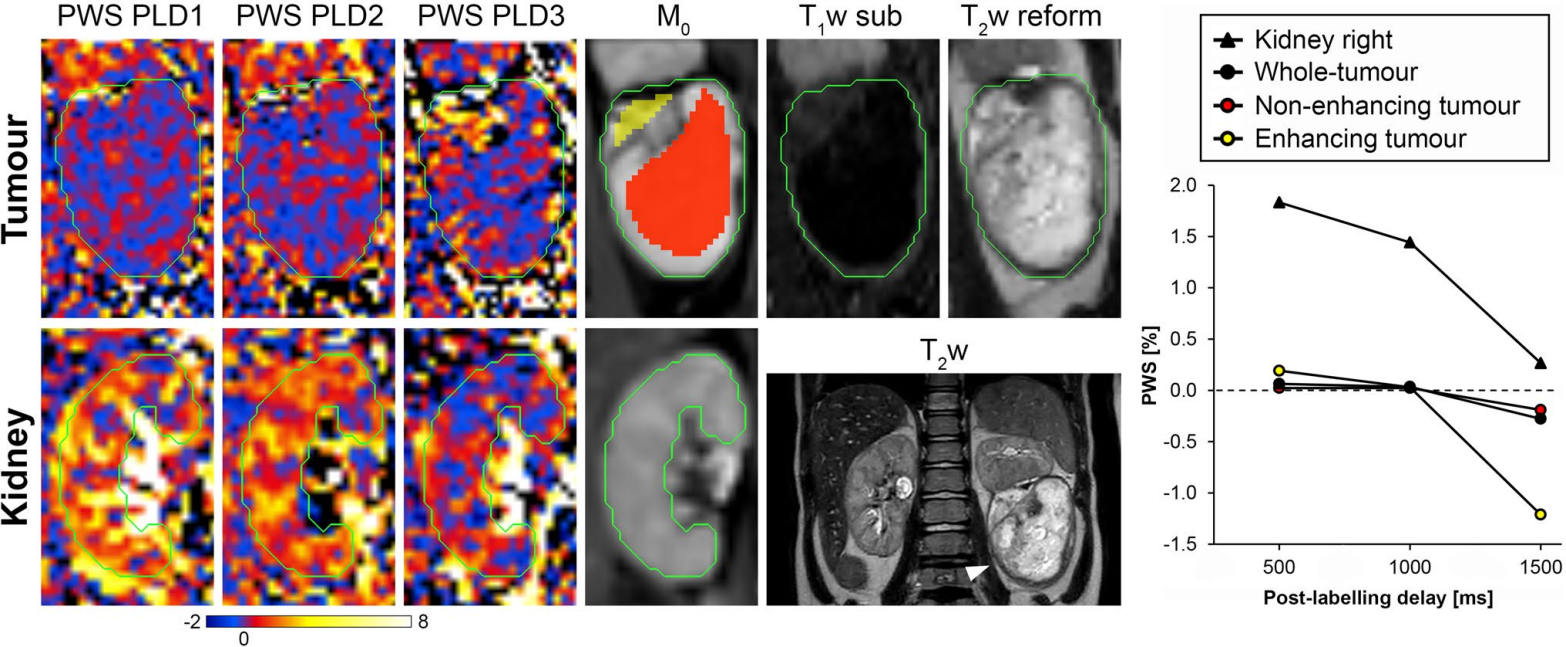
A 4-year-old male patient diagnosed with a bilateral nephroblastoma (subject 3 in Table 2). Processed ASL and M_0_ images are shown cropped around the tumour (upper row) and right kidney (bottom row), together with the reformatted T_2_w and T_1_w-subtracted images that were used for analysis of the tumour. The anatomical location of the nephroblastoma can be appreciated on the coronal T_2_w images (arrow indicates the largest tumour that was used for analysis). The graph on the right shows the perfusion-weighted signal (PWS) as a function of post-labelling delay (PLD) resulting from the ROI analysis. Green contours: whole-tumour and kidney regions that were used for the ROI analysis; yellow ROI (overlayed on M_0_ image tumour): tumour region with contrast enhancement on the T_1_w-subtracted image; red ROI (overlayed on M_0_ image tumour): tumour region without contrast enhancement on the T_1_w-subtracted image. Colour scale bar indicates PWS (∆M/M_0_ × 100%). PLD1 = 0.5 s; PLD2 = 1.0 s; PLD3 = 1.5 s

Image registration successfully reduced misalignment of M_0_ and raw ASL images, see example, Fig. 1, enabling further perfusion analysis in all subjects. For both analysed regions (tumour and kidney), on average 1 (range 0–3) label-control pair out of 10 per pCASL acquisition (delay time) was considered an outlier and was rejected. Examples of processed images that were used for analysis are shown in Figs. 2 and 3 for a neuroblastoma and nephroblastoma case, respectively. An overview of the ROI sizes used in the analysis for each tissue type are presented in Table 3. Due to the varying sizes of the tumours that is confounded by the age range of the patients, there was a large variation in the sizes of the ROIs.

**Table 3:** ROI sizes used in the analysis for each tissue type

ROI	Number of voxels		
	Mean	Minimum	Maximum
Whole-tumour	3568	231	9476
Contralateral kidney	1015	435	1575
Non-enhancing tumour	617	3	2422
Enhancing tumour	896	31	3718

### ASL PWS dynamics

**Fig. 4:**
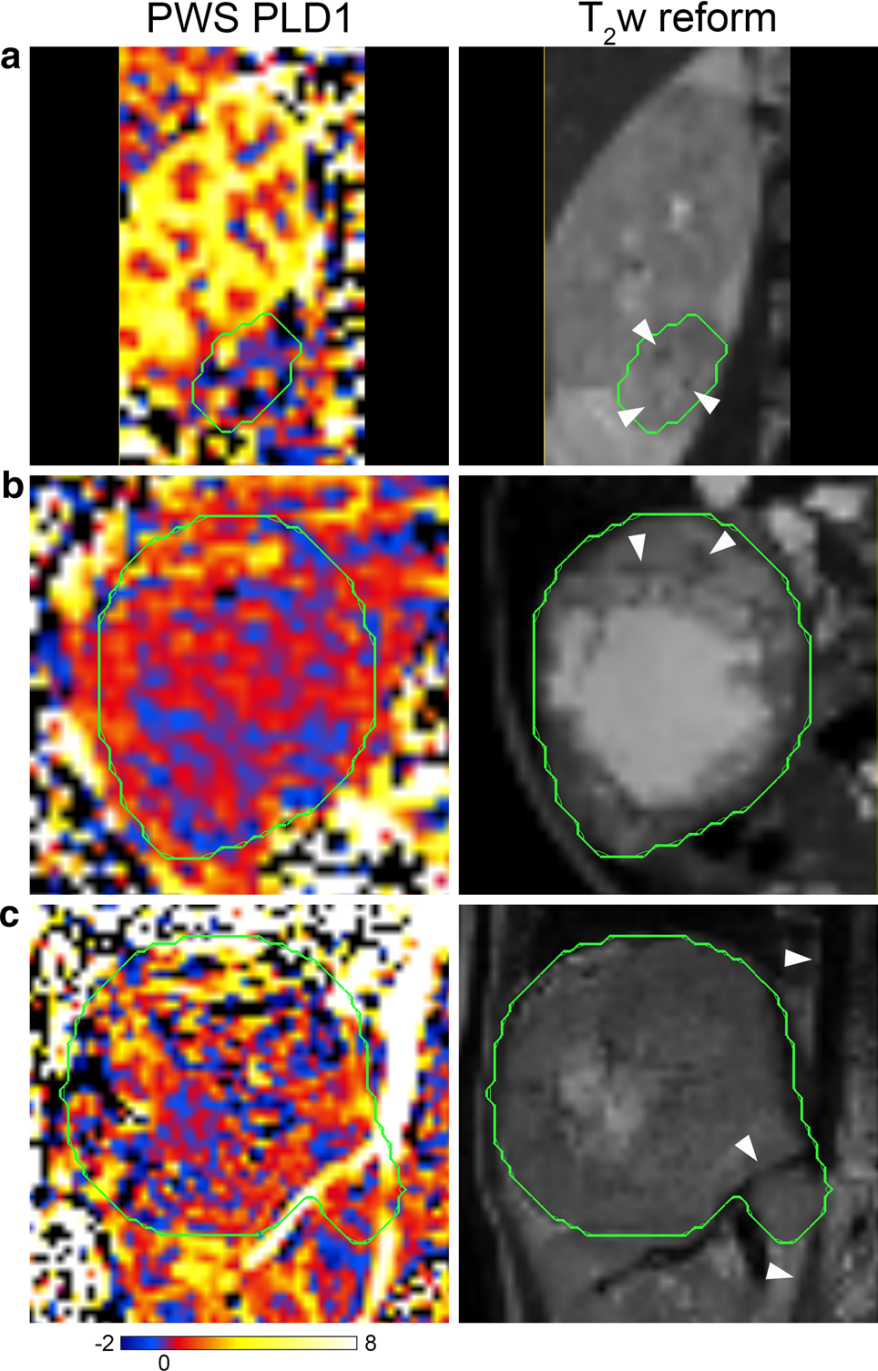
**a**, **b** Example cases from two patients (subject 2 and 9 in Table 2) showing high PWS inside the tumour on PWS images obtained with the shortest PLD of 0.5 s (PLD1) that coincided with local hypo-intense areas on the reformatted T_2_w images (white arrow heads). **c** This case (subject 8 in Table 2) shows very well the high PWS of labelled blood flowing inside the larger arteries (descending aorta and right renal artery; white arrow heads) indicating successful labelling. Many of the small regions with high PWS inside the tumour corresponded with hypo-intense regions on the T_2_w images, however, due to blurring effects after reformatting of the T_2_w image to the geometry of the ASL images these hypo-intense areas could not be visualised clearly here. The green contours indicate the tumour region. Colour scale bar indicates PWS (∆M/M_0_ × 100%)

A large variability in ASL PWS dynamics inside the tumour region was observed across the cases. The majority of tumours showed large regions with relatively low PWS intensity that remained stable over time for the three PLDs. Inside the tumour local regions with higher PWS intensity were observed that either increased or decreased with increasing PLD. Review of local regions with high PWS inside the tumour at the shortest PLD (0.5 s) that decreased over time indicated that these regions often coincided with local hypo-intense areas in the T_2_w images; examples are shown in Fig. 4.

#### Whole-tumour region vs (contralateral) kidney

**Fig. 5:**
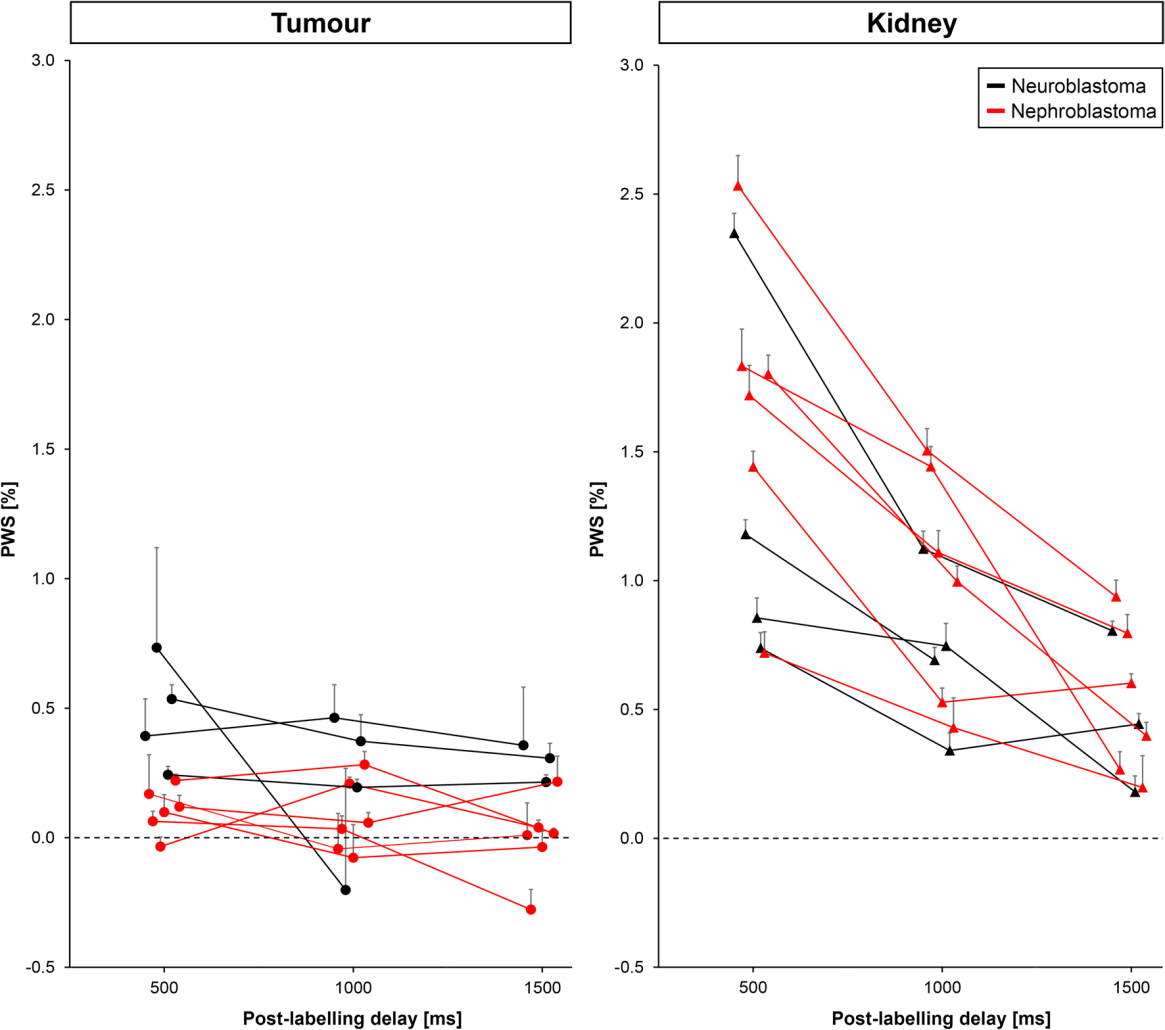
ROI analysis of PWS values obtained from pCASL images within the whole-tumour and contralateral kidney regions in 10 paediatric patients diagnosed with neuroblastoma (black) or nephroblastoma (red). The PWS measured in the kidney shows the method is sensitive to perfusion. Data points represent the average PWS in the whole-tumour region (left) and contralateral kidney region (right) at each delay time. The error bars represent the 95% confidence interval. For clarity data points are slightly shifted with respect to each other at each post-labelling delay and one-sided error bars are shown. *pCASL* pseudo-continuous arterial spin labelling, *PWS* perfusion weighted signal

PWS values obtained from pCASL images as a function of PLD within the whole-tumour and kidney regions are shown in Fig. 5. Mean whole-tumour PWS was 0.25 ± 0.22% (range − 0.034 to 0.73%), 0.13 ± 0.20% (range − 0.20 to 0.46%), and 0.094 ± 0.19% (range − 0.28 to 0.36%) for PLDs of 0.5, 1.0 and 1.5 s, respectively, vs 1.5 ± 0.61% (range 0.72–2.5%), 0.89 ± 0.39% (range 0.34–1.5%), and 0.51 ± 0.27% (range 0.18–0.94%) in the contralateral kidney parenchyma region. In general, the mean PWS within the whole-tumour region was low in all subjects. For neuroblastoma cases, the mean whole-tumour PWS appeared to be slightly higher than the nephroblastoma cases.

#### Non-enhancing vs enhancing tumour regions

**Fig. 6:**
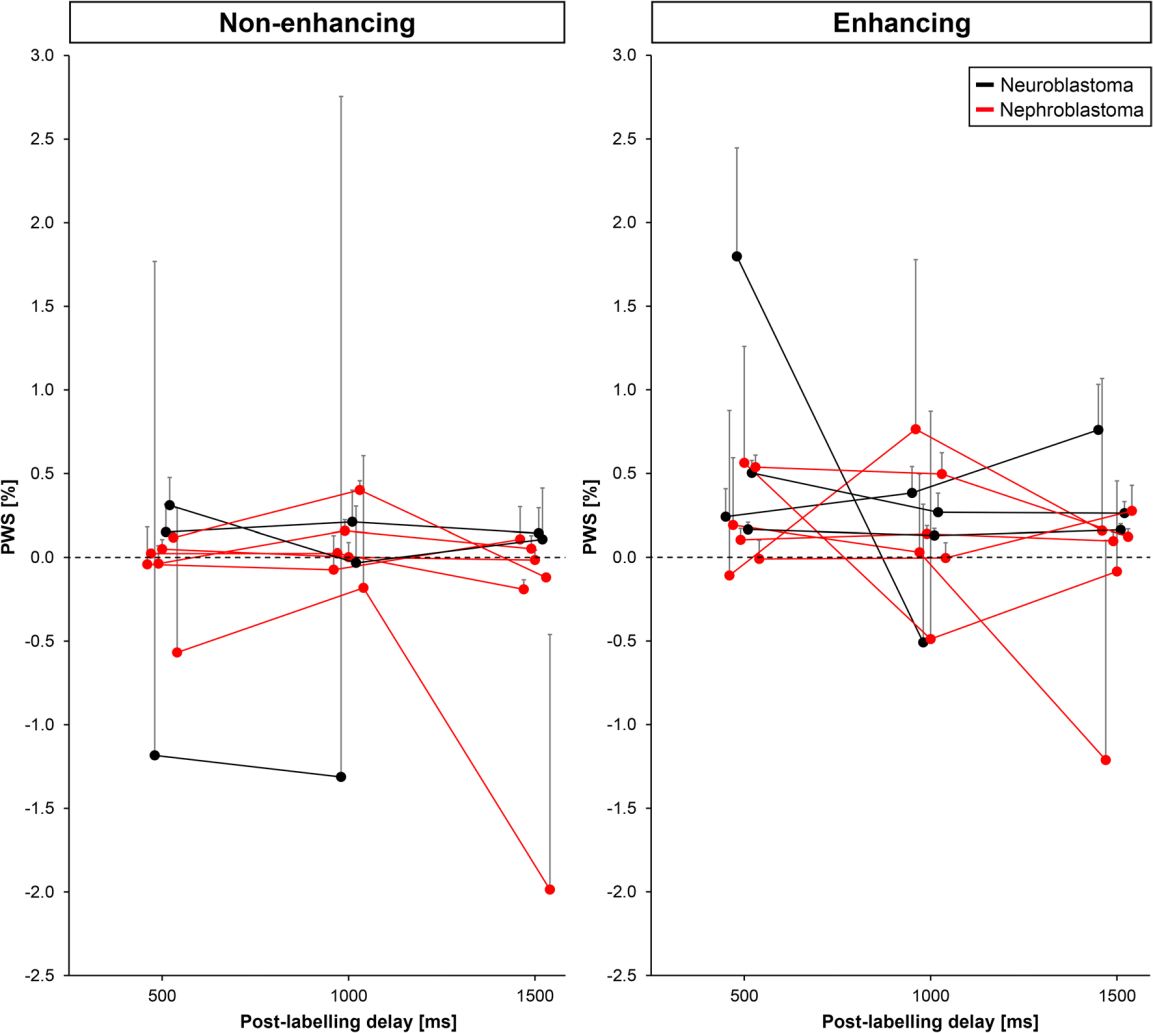
ROI analysis of PWS values obtained from pCASL images within non-enhancing and enhancing subregions of the tumour in 10 paediatric patients diagnosed with neuroblastoma (black) or nephroblastoma (red). Data points represent the average PWS in non-enhancing (left) and enhancing tumour regions (right) at each delay time. A minority of two neuroblastoma cases show increased PWS in enhancing regions inside the tumour. One of the neuroblastoma patients is missing a data point (post-labelling delay 1500 ms), since this scan was accidentally omitted during acquisition. The error bars represent the 95% confidence interval. For clarity data points are slightly shifted with respect to each other at each post-labelling delay and one-sided error bars are shown. *pCASL* pseudo-continuous arterial spin labelling, *PWS* perfusion weighted signal

Mean PWS values obtained from pCASL images as a function of PLD within non-enhancing and enhancing subregions of the tumour after contrast administration are shown in Fig. 6. Mean PWS was − 0.13 ± 0.46% (range − 1.2 to 0.31%), − 0.089 ± 0.49% (range − 1.3 to 0.40%), and − 0.24 ± 0.72% (range − 2.0 to 0.14%) in non-enhancing tumour regions, and 0.40 ± 0.51% (range − 0.11 to 1.8%), 0.12 ± 0.38% (range − 0.51 to 0.76%), and 0.060 ± 0.50% (range − 1.2 to 0.76%) in enhancing tumour regions for PLD 0.5, 1.0 and 1.5 s, respectively. Overall, non-enhancing tumour regions consistently corresponded with measured PWS around zero. For the enhancing tumour regions, there were two neuroblastoma cases with clearly higher mean PWS values, but most other cases showed very low PWS, similar to that observed in the non-enhancing regions.

## Discussion

We conducted a study to establish the feasibility of ASL-MRI in paediatric neuroblastoma and nephroblastoma. The study showed that this was feasible within 5 min of extra scan time. After realignment, the acquired ASL images were usable for analysis in a paediatric population under non-invasive ventilation and free-breathing conditions. The perfusion signal in the kidney indicates that labelling was successfully performed in all subjects, yet, the majority of the included abdominal tumours presented with relatively low PWS. Within the tumours, regions with higher PWS were observed, either related to labelled blood inside vessels (at short PLD) or to labelled blood accumulating inside tumour tissue over time (increasing with PLD). Conversely, comparison of ASL perfusion-weighted image findings with T_1_w enhancement after contrast administration showed that regions lacking contrast enhancement also showed PWS around zero.

The ASL scan was obtained in a clinical setting. This defined our choice for the pCASL labelling approach, since this method was clinically available on our MR system. The pCASL labelling approach is currently recommended for brain and kidney perfusion imaging in adults [16, 19]. pCASL requires the separate planning of a labelling slab at the location of the feeding arteries that must be positioned perpendicular to the blood flow direction to ensure high labelling efficiency. Since each abdominal tumour is different in size, shape, composition and location, the feeding vessels of each tumour are unique. These tumour feeding arteries are often tortuous with varying length and difficult to locate [20]. Ambiguity of the feeding vessel location forced us to place the labelling slab perpendicular to the descending aorta to ensure blood was labelled before entering the tumour. This location was feasible for planning of the labelling slab in all patients, but will in some patients imply placement above the diaphragm at the level of the lungs which may negatively influence labelling efficiency [21]. Tortuosity and length variation of the feeding vessels induce variability in the arterial transit time of labelled blood and the amount of label accumulation in tissue that can take place during the PLD [22]. Increasing PLD to allow time for the labelled blood to travel from the aorta into the tumour and accumulate inside the tumour tissue will limit the available PWS due to T_1_ decay. Since no previous ASL studies on abdominal tumours in children have been reported yet, and hemodynamics and tumour characteristics can be very different from those in adults [14], ASL data was acquired with three different PLDs to evaluate the influence of inflow time on the perfusion signal. In the current study, high PWS was already observed at the first PLD of 0.5 s in the renal cortex, which seemed to reduce rapidly with increasing PLD (as shown in Fig. 5). This arrival and decay of renal PWS appears to be faster than reported in adults [16], underlining the difference in hemodynamics between children and adults. Other factors such as variability in blood and tissue T_1_ values that are known to be present between children, and an age-dependency of the arterial transit time of blood flowing from the labelling plane to tissue of interest also have an influence on the measured PWS [23–26].

In general, the whole-tumour mean PWS was relatively low in all patients, especially compared with the contralateral kidney region (as shown in Fig. 5). Kidneys are normally well-perfused, so high PWS was expected in this organ. Therefore, the measured PWS values in the contralateral kidney region served as an indication that label was created and perfusion signal could be measured using this ASL method, especially since the measured PWS in the tumours was in general relatively low. The usage of large ROIs covering the entire tumour (see examples in Figs. 2 and 3) may have obscured local regions of high PWS inside the larger areas of low PWS in the calculation of the mean PWS averaged over all voxels inside this whole-tumour region. Review of local tumour regions with high PWS at the shortest PLD that decreased over time indicated that these regions often coincided with local hypo-intense areas on the T_2_w images (as shown in Fig. 4). These type of local hypo-intense areas on T_2_w images are usually caused by flow void effects inside a blood vessel. Small local areas of high PWS at the shortest PLD are, therefore, likely to reflect presence of tumour vasculature. On the other hand, a local tumour region with high PWS that increases or remains more or less constant over time may be an indication of tissue perfusion. Validation of in vivo perfusion measurements are in general difficult to perform, due to the lack of a gold standard technique.

The comparison of tumour contrast enhancement on T_1_w-subtracted images with the ASL perfusion-weighted image findings was intended to assess which aspects of tumour physiology are being measured with the applied ASL method, and whether these are physiologically plausible. In principle, the methods do not measure the same physiological processes: contrast enhancement is the result of either blood flow or vascular leakage, where ASL PWS is the result of blood flow only. Non-enhancing tumour regions (no blood flow nor leakage) should, therefore, correspond with PWS around zero (no blood flow), which was the case for all non-enhancing tumour regions in this study. Enhancing tumour regions, on the other hand, were more difficult to compare directly with ASL findings in the same tumour regions. Most tumours showed very low PWS inside enhancing regions (as shown in Fig. 6). This could either mean that the enhancement was mainly driven by vascular leakage, which usually builds up over time, or the arterial transit time of the labelled blood was too long to reach the tumour tissue and accumulate. Interestingly, there were two neuroblastoma cases with clearly higher PWS values in the enhancing regions, indicating a possible blood flow effect. It could also be that the arterial transit time of the labelled blood was much shorter for these two tumours, making the accumulation of PWS better visible.

Thus far, ASL MRI in the body/abdomen has mainly been applied for renal perfusion imaging in adults. Recently, a consensus-based paper describing technical recommendations for clinical translation of renal ASL MRI has been published [16], that was used as a starting point for setting up the current study. For the kidneys, Echeverria-Chasco et al. [27] recently showed that the labelling efficiency and robustness of pCASL for renal applications can be optimized and depends on the aortic blood flow velocities. A similar optimization of gradient parameters for blood flow velocities in a paediatric population could be performed. However, this does not resolve the problem of heterogeneity in tumour location and feeding vessels between patients, that influence the transit-time of labelled blood from the labelling plane to the tumour tissue. This currently makes it difficult to identify the cause of low PWS inside the tumour that remains stable across all PLDs (as shown in Fig. 5) that could either be due to low or late perfusion of the tumour. It is, therefore, very important that labelling is performed as close as possible to the anatomy of interest. A flow-based labelling approach, such as velocity-selective ASL (VSASL) [28, 29] and velocity-selective-inversion prepared ASL (VSI-ASL) [30], that is insensitive to transit-time and does not require the planning of a separate labelling slab, may provide a promising alternative to spatially selective ASL techniques for tumour perfusion imaging of paediatric abdominal tumours. Recently, a few studies have already shown promising results of flow-based ASL imaging in the abdomen for the kidneys [31, 32] and placenta [33, 34] in adults. Another recent study in paediatric patients with Moyamoya has shown VSASL offers a powerful approach to accurately image perfusion in these patients due to transit delay insensitivity [35]. On the other hand, due to the wide availability of pCASL on clinical scanners, continuing with this labelling approach may still be a good idea, possibly after adaptation/optimization for blood flow velocities in children.

This study has limitations. A first limitation of this study is that the number of patients included was relatively small with heterogeneous characteristics, which are, however, usual limitations inherent to the nature of an explorative study on retrospective data. In general, paediatric abdominal tumours are very heterogeneous with diverse pathological features, and the number of patients for each tumour type are often relatively small. This poses challenges for conducting research with this population. So far, feasibility of ASL has been primarily studied in the brain reflecting the difficulty in evaluating new techniques in paediatric patients with rare diseases. Second, the retrospective nature of this study in a clinical setting limited the amount of time allowed for additional scans that could be performed. This meant that more extensive evaluation that would require additional scans, such as B0 and B1 maps or labelling efficiency measurements, to evaluate the technical validity of the ASL measurements or a repeatability analysis could not be performed. A future study with a prospective setup would, therefore, be a logical next step. Default implemented pCASL labelling settings were used, which have been optimised for brain application in adults. Optimisation of pCASL labelling parameters for blood flow characteristics of the descending aorta in a paediatric population may improve labelling efficiency and reduce labelling variation between patients, as has been shown for renal application in adults [27]. In ASL, the labelling efficiency is mostly assumed as a constant parameter in the kinetic model for perfusion quantification [16, 19, 36]. In fact, in adults labelling efficiency variation may be several tens of present for settings not optimised for the abdomen [27]. In the paediatric population, which is relatively heterogeneous and with many factors that could potentially have an influence on the measured PWS, a labelling efficiency measurement on a subject basis may provide insight in the variability in this population. Third, most patients were under anaesthesia during scanning. Sedatives may have impact on tissue perfusion, complicating the interpretation of perfusion data [24, 26]. The general effects of sedatives on ASL imaging are currently unknown, and remain an important factor to consider in future larger studies of paediatric imaging. Finally, due to the explorative nature of this study, ASL-MRI was only performed at a single visit in each patient, either at initial staging or during follow-up. Possible changes in tumour perfusion during treatment could, therefore, not be assessed. This should be further investigated in future studies by measuring ASL at multiple visits during treatment within the same patient.

In conclusion, this study demonstrated the feasibility of ASL-MRI with a pCASL labelling approach in paediatric patients with solid abdominal tumours. In this population considerable differences in PWS were observed both within the individual tumour and between subjects, with on average sixfold lower PWS in the whole-tumour region compared with the contralateral kidney. Within the tumour, non-enhancing tumour regions consistently corresponded with PWS around zero, whereas some enhancing tumour regions clearly showed higher mean PWS values. The preliminary data presented here provides a basis for further research on non-invasive perfusion measurements of solid abdominal tumours using ASL-MRI, specifically in paediatric patients. Further research should be directed towards improving the labelling approach, for instance by optimizing pCASL labelling settings or by exploring other labelling approaches, such as flow-based ASL techniques.
