# Correction to: Perfusion imaging of neuroblastoma and nephroblastoma in a paediatric population using pseudo‑continuous arterial spin‑labelling magnetic resonance imaging

**DOI:** 10.1007/s10334-021-00955-8

**Published:** 2021-09-01

**Authors:** Anita Harteveld, Annemieke Simone Littooij, Max Maria van Noesel, Marijn van Stralen, Clemens Bos

**Affiliations:** 1 Department of Radiology, University Medical Centre Utrecht, Utrecht University, P.O. box 85500, 3508 GA Utrecht, The Netherlands; 2 Department of Radiology and Nuclear Medicine, Erasmus MC University Medical Centre Rotterdam, Rotterdam, The Netherlands; 3 Princess Máxima Centre for Paediatric Oncology, Utrecht, The Netherlands

## Correction to: Magnetic Resonance Materials in Physics, Biology and Medicine 10.1007/s10334-021-00943-y

The original version of this article unfortunately contained a mistake. First author name was incorrectly written as Anita Adriaantje Harteveld.

Author name should be Anita Harteveld.

